# Scientometric Analysis and Research Mapping Knowledge of Coconut Fibers in Concrete

**DOI:** 10.3390/ma15165639

**Published:** 2022-08-16

**Authors:** Mingli Gu, Waqas Ahmad, Turki M. Alaboud, Asad Zia, Usman Akmal, Youssef Ahmed Awad, Hisham Alabduljabbar

**Affiliations:** 1 Inner Mongolia Vocational and Technical College of Communications, Chifeng 024005, China; 2 Department of Civil Engineering, COMSATS University Islamabad, Abbottabad 22060, Pakistan; 3 Civil Engineering Department, College of Engineering and Islamic Architecture, Umm Al-Qura University, P.O. Box 5555, Makkah 21955, Saudi Arabia; 4 Department of Concrete Structures and Bridges, Slovak University of Technology in Bratislava, 811 07 Bratislava, Slovakia; 5 Department of Civil Engineering, University of Engineering and Technology, Lahore 39161, Pakistan; 6 Structural Engineering Department, Faculty of Engineering & Technology, Future University in Egypt, New Cairo 11835, Egypt; 7 Department of Civil Engineering, College of Engineering in Al-Kharj, Prince Sattam Bin Abdulaziz University, Al-Kharj 11942, Saudi Arabia

**Keywords:** coconut fiber, concrete, cementitious composites, scientometric analysis

## Abstract

Biodegradable materials are appropriate for the environment and are gaining immense attention worldwide. The mechanical properties (such as elongation at break, density, and failure strain) of some natural fibers (such as Coir, Hemp, Jute, Ramie, and Sisal) are comparable with those of some synthetic fibers (such as E glass, aramid, or Kevlar). However, the toughness of coconut fibers is comparatively more than other natural fibers. Numerous studies suggest coconut fibers perform better to improve the concrete mechanical properties. However, the knowledge is dispersed, making it difficult for anyone to evaluate the compatibility of coconut fibers in concrete. This study aims to perform a scientometric review of coconut fiber applications in cementitious concrete to discover the various aspects of the literature. The typical conventional review studies are somehow limited in terms of their capacity for linking different literature elements entirely and precisely. Science mapping, co-occurrence, and co-citation are among a few primary challenging points in research at advanced levels. The highly innovative authors/researchers famous for citations, the sources having the highest number of articles, domains that are actively involved, and co-occurrences of keywords in the research on coconut-fiber-reinforced cementitious concrete are explored during the analysis. The bibliometric database with 235 published research studies, which are taken from the Scopus dataset, are analyzed using the VOSviewer application. This research will assist researchers in the development of joint ventures in addition to sharing novel approaches and ideas with the help of a statistical and graphical description of researchers and countries/regions that are contributing. In addition, the applicability of coconut fiber in concrete is explored for mechanical properties considering the literature, and this will benefit new researchers for its use in concrete.

## 1. Introduction

Typical cementitious concrete is usually excellent under compressive loading but weak under the application of tensile forces. However, steel reinforcement is incorporated if the concrete structure is intended to bear tensile forces. The steel reinforcement overcomes the said deficiency in concrete, and the respective concrete is known as reinforced cementitious concrete. The typical brittle nature of cementitious concrete can be altered by having effective modifications in it [1,2]. Therefore, the addition of different types of fibers was proposed with the passage of time to overcome this issue [3]. Moreover, in the 1960s, the concept of fibers addition in cementitious concrete was initiated in which short dispersed fibers were used for incorporation into concrete to enhance the tensile strength of concrete by decreasing its brittleness, ultimately resulting in a particular concrete type named fiber-reinforced concrete [4,5]. The incorporation of fibers such as metallic/steel fibers [6,7,8], natural fibers [9,10], synthetic fibers [11,12,13], and mineral fibers [2,8,14] in concrete is performed [15] for enhancing the toughness behavior of concrete [16,17]. The concept of fiber spacing theory for fiber-reinforced concrete (FRC) was initially introduced by Batson [18]. Among secondary/alternative materials that aid in developing eco-friendly cementitious concrete [19,20,21], natural/agricultural/plant fibers come out as the most effective dispersed reinforcing materials for achieving sustainable [22] and environment-friendly [23] development. Gangil et al. [24] conducted a comparative study of properties of natural and synthetic fibers and concluded that the values for elongation at break (%), failure strain (%), and density (g/cm^3^) of Coir, Hemp, Jute, Ramie, and Sisal fibers are in line with the respective properties of E-glass and aramid or Kevlar fibers. Moreover, the availability of natural fibers is worldwide, which cost very little compared to artificial fibers [25]. In the last several decades, the use of natural fibers has been gaining the attention of an increasing number of scientists, researchers, and academics for having alternative economical and eco-friendly materials compared to artificial/synthetic fibers in the last several decades [26,27]. Flax, wheat straw, jute, rice straw, kenaf, sugarcane bagasse, coir, bamboo, banana husk, ramie, and henequen [28,29,30,31] are some natural fibers.

**Figure 1:**
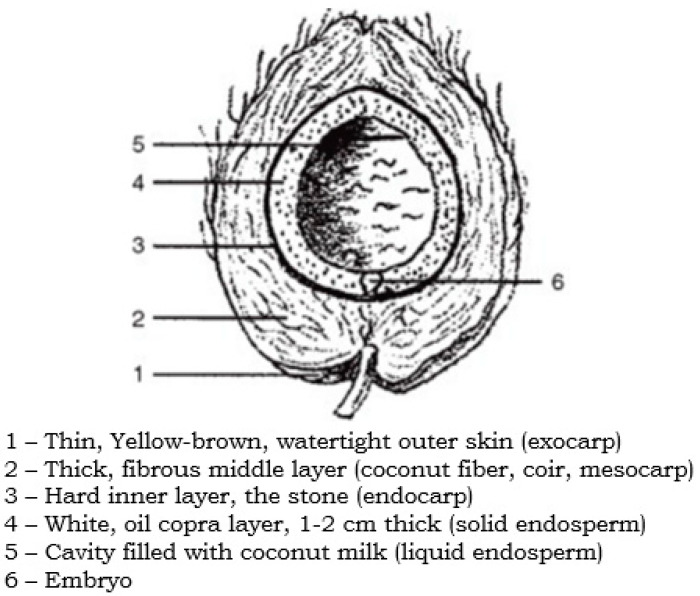
Coconut longitudinal section (adapted from [37]).

**Figure 2:**
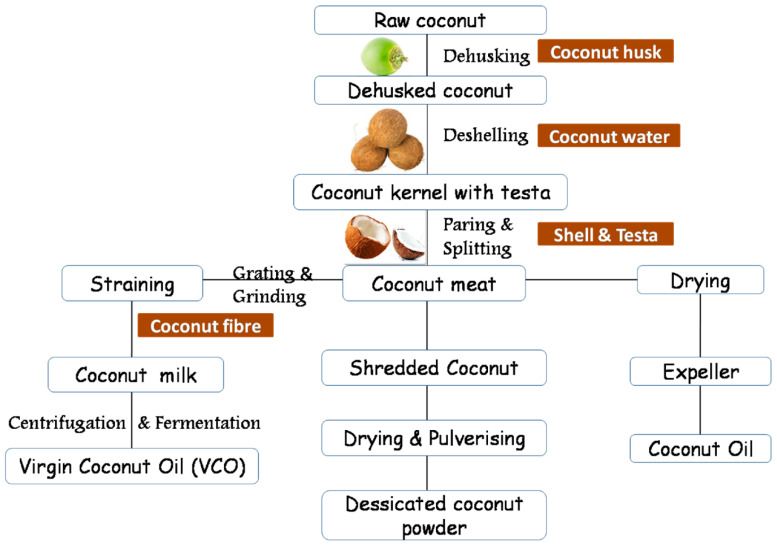
Stepwise procedure for extracting fibers and other products from coconut (adapted from [47]).

Among all natural fibers, coconut fiber is a famous one that is extracted from the coconut fruits husks (Figure 1) and is utilized for developing long-lasting and high-strength products [32]. Globally, coconut is cultivated in multiple countries, specifically in subtropical and tropical areas that contribute significantly to economic growth. In an urge to enhance the mechanical properties of concrete, coconut fiber, as a dispersed reinforcement, has been the focus of multiple studies [33,34,35]. Coconut fiber (Coir) is mainly classified into four different kinds such as buffering coil, bristle coil, white fibers, and brown fibers. The most general form of coconut fiber is bristle coil, having little or no content of pulp and not less than five inches. Brown fiber is extracted from the mature coconut, which is conventionally highly thick, strong, and abrasive, making it the most suitable and widely used fiber [36]. Distinct from brown fiber, which is extracted from mature coconut, the extraction of white fiber is conducted from immature coconut and, usually, it is not as durable as the brown fiber is [37]. The whole stepwise procedure for extracting fibers and other products from coconut is illustrated in Figure 2. Coconut fiber has a bulk quantity of lignin and a lower quantity of cellulose, making it versatile, strong, and solid [38]. The length, diameter, and aspect ratio of coconut fibers is 8–250 mm [39,40,41], 0.25–1.00 mm [39,42,43], and 100 [42], respectively. The reported ranges for the density and water absorption of coconut fiber in the literature are 0.67–10 g/cm^3^ [43] and 130–180% [44], respectively. Furthermore, the reported ranges for the tensile strength and elongation of coconut fiber in the literature are 15–405 MPa [40,42,44] and 25–75% [39,40,44], respectively. Regarding the mechanical properties of coconut-fiber-reinforced concrete, Khan and Ali [45] studied the effect of 50 mm long coconut fibers having 2% content, by cement mass, in concrete. The study was concluded with enhanced mechanical properties. Similarly, Khan et al. [46] reported the enhanced/improved energy absorption and toughness index of coconut-fiber-reinforced concrete with respect to the control specimen.

However, due to the organic/biodegradable nature of coconut fibers, their durability is still a concern. Hence, efforts are made to enhance the durability of coir either by soaking it in hot water or some chemical solutions [48]. Accordingly, coconut fibers are used and available in the two types, i.e., untreated and treated. Ramli et al. [41] determined the properties of concrete having untreated coconut fibers under different ageing conditions such as exposure to air and seawater. The chloride penetration, intrinsic permeability, and carbonation depth tests were performed to evaluate its durability. The study concluded with coconut-fiber-reinforced concrete′s enhanced strength and durability. Moreover, it was recommended in the study that treatment should be applied to coconut fibers before incorporating them in concrete for providing it with protection from degradation. According to one more study [49], upon treatment of coconut fibers, removing pectin, lignin, hemicellulose, and wax from the surface of the fiber would result in parenchyma cells elimination, which enhances the contact area among globular marks and fibrils. As a result, the enhancement in fiber roughness is caused, which ultimately enhances the adhesion among the fibers and matrix [49]. Furthermore, coconut fibers have a lesser conductivity of heat; however, being stiff and strong, the tensile, compressive, and flexural strengths of concrete enhance but with reduced concrete weight [50].

The increasing environmental problems have led to the development of research on environmentally friendly construction materials such as coconut-fiber-reinforced concrete. Several researchers studied coconut fiber in concrete instead of artificial/synthetic/steel fibers. However, the research knowledge on coconut-fiber-reinforced concrete is still scattered, and there is no easy way to assess the importance of coconut-fiber-reinforced concrete. The difficulties in creative investigation and scholarly collaboration are aroused due to the information limitations of researchers. For this purpose, establishing and employing a technique for scientists/researchers to acquire the necessary information from reliable sources is essential. To the best of the author’s knowledge, no scientometric review has yet been conducted on the literature regarding the utilization of coconut fibers in concrete. Hence, employment of a scientometric method with the help of a software tool can assist in overcoming this gap. The fundamental purpose of this research is to provide an in-depth literature review on incorporating coconut fibers in cementitious composites, focusing on its mechanical properties, applications, and current state in the construction industry. The research gaps and challenges in applying coconut-fiber-reinforced concrete are also elaborated in the present work. The scientometric analysis of published research in coconut-fiber-reinforced concrete up to 2022 is aimed in the current study. The bulk research database may be assessed quantitatively by undertaking scientometric analysis with the help of a suitable software tool. The conventional review-based research is weak to some extent in its capacity for connecting numerous segments of the literature wholly and precisely. Co-citation, science mapping, and co-occurrence are a few main investigation parameters in the modern era [51,52,53]. The discovery of sources having keywords co-occurrence, the primary authors as per articles and citations, vigorously involved research zones, and the most research publications in coconut-fiber-reinforced concrete may also be made with the help of scientometric analysis. The Scopus database is utilized to extract the bibliometric dataset of 235 relevant research publications. The current study would assist academics of the engineering field belonging to various geographical locations in exchanging ground-breaking novel methods/ideas, creating joint ventures, and forming research alliances due to the graphical and statistical depiction of countries and authors. Furthermore, evaluating and critically summarizing the review data on coconut fibers in concrete through this scientometric analysis, the industrial experts of this field can gain a comprehensive insight and clear picture regarding the coconut-fiber-reinforced concrete. This analysis would further allow the relevant industrial experts to gain an understanding of the available knowledge on coconut fibers in concrete, as well as the limitations and boundaries of this sustainable material prior to its practical implementation.

## 2. Methodology

The scientometric analysis is performed in the current study for the research database to determine the various features of bibliographic data [54,55,56]. Multiple research studies have been reported and carried out in said area showing the uncertain employment of well-known search engines. Scopus and Web of Science, the two more accurate search engines, are mainly discovered for the stated aim [57,58]. We collect research data on coconut fibers in concrete for conducting this research using Scopus, a highly suggested academic search engine [59,60]. Today, the search in Scopus for “coconut fiber reinforced concrete” finds 235 documents from 2010 to 2022. Numerous preferences-based filters are employed for evading the data, which is unnecessary. “Journal review”, “journal research article”, “conference paper”, and “conference review” are adopted as the type of documents. “Journal” and “conference proceeding” are taken as “source type”. The period selected as “publication year” is “2022”, and “language” is selected as “English”. Further refinement is conducted by selecting “subject areas” such as “material science”, “engineering”, and “environmental science”. With the application of the above-mentioned refinements, a total of 235 records are taken. In the same manner, several research studies have been performed by applying the same technique [61,62,63].

**Figure 3:**
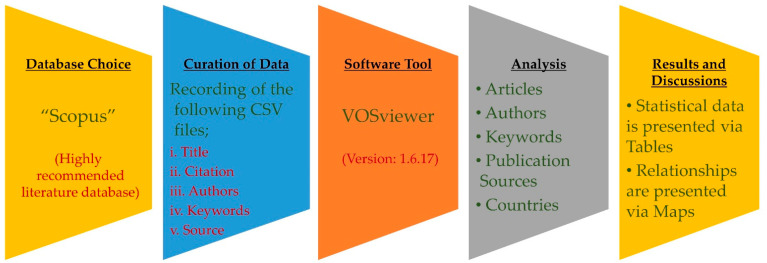
Research methodology sequence.

In the academic field, the bibliometric data are analyzed by developing scientific mapping that is generally applied for analyzing scientometric investigations [64]. Using an appropriate software tool, Scopus records are saved using Comma-Separated Value (CSV) files for evaluation. The quantitative determination of the scientific visualization and the recovered records literature is developed using VOSviewer (version: 1.6.17). VOSviewer is a highly proposed and widely applied tool in the field of academics over a larger range of research areas, and this open-source mapping has instant accessibility [65,66,67,68]. Hence, the VOSviewer application in this research justifies its aims. Further assessment is conducted by loading the attained CSV files in VOSviewer for data integrity and consistency. Further, the evaluation is also made for the participation of countries, highly cited researchers with significant publications, bibliographic data, frequent keywords, and sources of publication. The several aspects, along with their relationships and co-occurrence, are also presented graphically; however, the statistics of figures are provided in tables. The procedural flowchart for conducting scientometric analysis is illustrated in Figure 3.

## 3. Results Analysis

### 3.1. Annual Publications and Related Subject Areas

**Figure 4:**
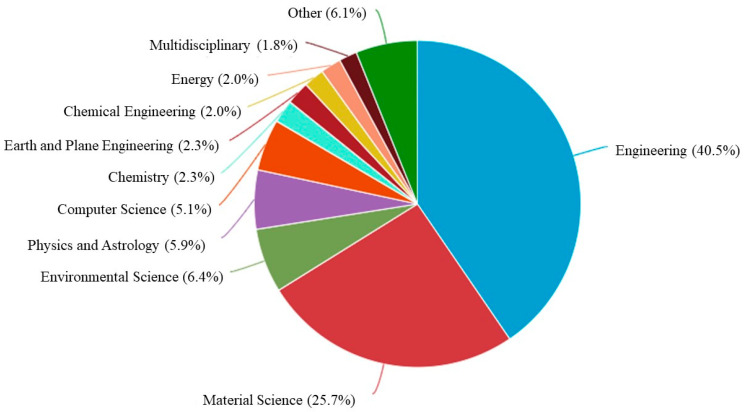
The subject area of articles.

**Figure 5:**
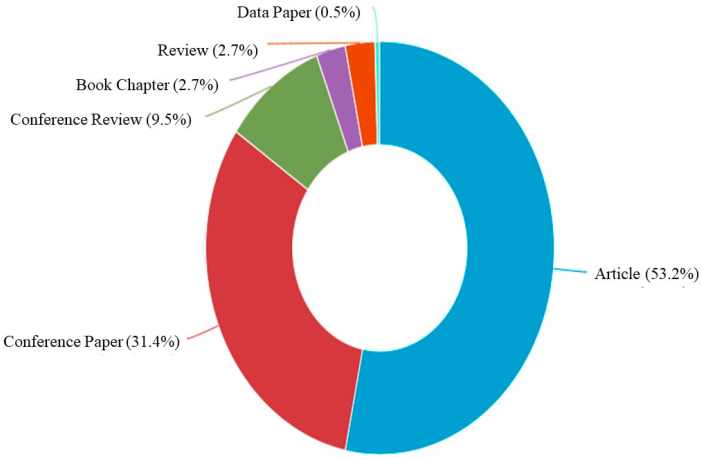
Various types of documents published in the related study field.

**Figure 6:**
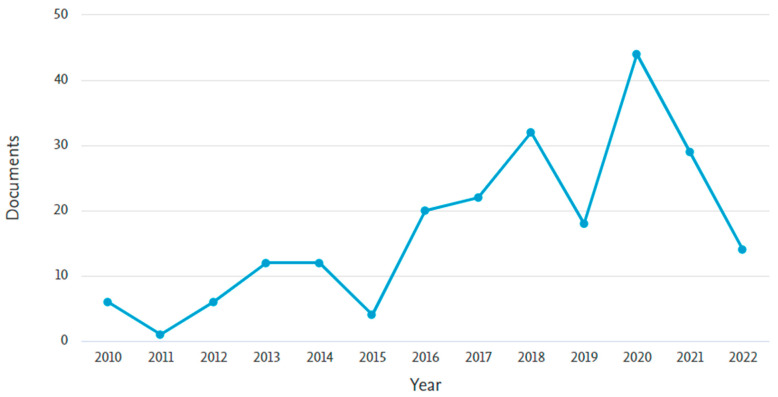
Annual publication trend of articles.

A Scopus analyzer is employed for the analysis to extract the most relevant research areas. Figure 4 depicts that engineering with almost 41%, material sciences with 26%, and environmental science with 7% come out as three sections producing leading articles and contributing 73% overall as per document count. Additionally, the identification of the type of articles for the searched terms in the Scopus database is provided in Figure 5. As per the findings of this study, out of all the documents, there are almost 53% journal, 31% conference, and 10% conference review articles. Figure 6 represents the current research area’s annual publication trend from 2010 to 2022. It is also noteworthy that the first article in this field appeared in 2010. Initially, the trend fell in 2011. However, after 2017, there were almost twenty or more publications each year with significant enhancement up to 45 publications in 2020. The publications for the current year are also represented in the same plot to show the current trend of publication growth in the considered research field until the mid of this year, i.e., 2022. The number of publications in 2022 until today indicates that a significant rise is expected in the research on coconut fibers in cementitious concrete by the end of this year.

### 3.2. Sources of Publications

**Figure 7:**
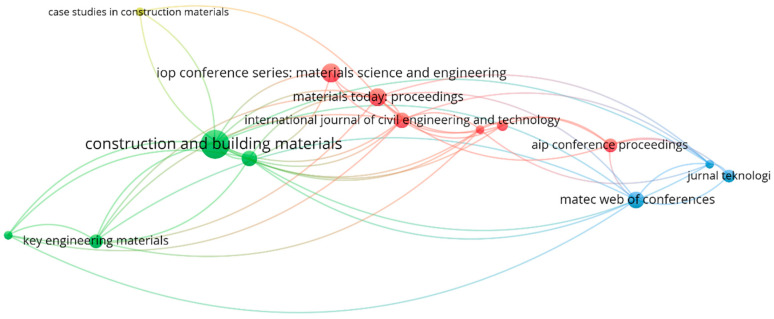
Scientific visualization of publication sources in the related research area.

The sources of publication are determined from the collected bibliographic data using VOSviewer. “Unit of analysis” is taken as “sources”, whereas “kind of analysis” is opted as “bibliographic coupling” during the analysis. Table 1 illustrates the 14 sources for 235 publications depicting research data on coconut fibers in concrete until 2022 and the number of citations. “Construction and Building Materials” with 21 papers, “IOP Conference Series: Materials Science and Engineering” with nine articles, and “Materials Today: Proceedings” with eight articles are revealed as three primary sources/journals, based on the paper count. Moreover, from a citation point of view, the three primary sources are “Construction and Building Materials”, “Materials”, and “Materials Today: proceedings”, having 876, 122, and 56 citations, respectively. This considerable research revealed in the field of coconut fiber in concrete became the aim for conducting this scientometric analysis. Further, the already conducted typical review research is insufficient for establishing the scientific visualization maps. The journals’ mapping with at least two articles in the considered field of research is illustrated in Figure 7. The number of research articles in the “coconut fiber reinforced concrete” area is depicted by the circle size representing the journal’s impact. The higher the circle’s dimension, the more superior the effect. It is observed that the circle with the highest dimension is for “Construction and Building Materials”, depicting the considerable source impact in the relevant research field. Depending upon the type, four groups are established that are shown by four different colors, i.e., green, red, blue, and yellow. This group formation is made with respect to the frequency of occurrence of co-citations for similar articles [69]. In VOSviewer, groups are created based on patterns of co-citation for published articles. Six sources in the red group show recurrent co-citations in similar works. Furthermore, the spacing between journals/frames in a single group represents considerable relationships than other far-spaced frames. As an illustration, “Materials Today: proceedings” is closely correlated with “International Journal of Civil Engineering and Technology” as compared to “IOP Conference Series: Materials Science and Engineering”.

### 3.3. Keywords

**Figure 8:**
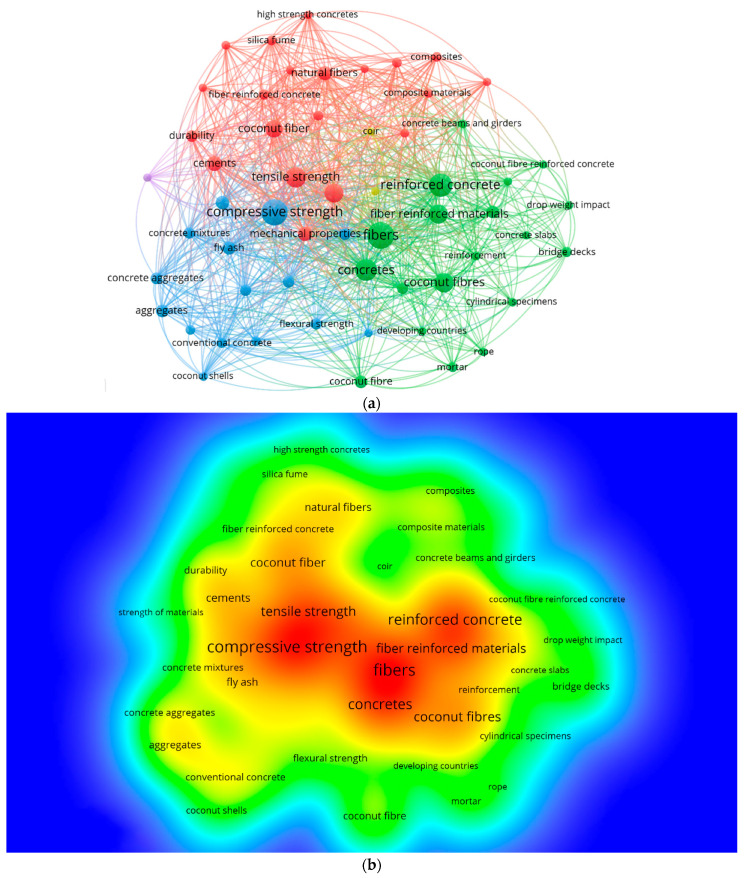
Keywords analysis: (**a**) Scientific visualization. (**b**) Density visualization.

The vital focus of a research domain is elaborated and defined by the keywords [70]. For the assessment, “co-occurrence” is selected as “analysis type”, and “all keywords” is adopted as “analysis unit”. The limitation for the most minor repetition is set to 20 for a single keyword. In Table 2, the thirty most widely used leading keywords in published articles on considered areas of research are listed. Fibers, concretes, compressive strength, reinforced concrete, tensile strength, and coconut fibers are the six keywords that have been employed most frequently for the coconut-fiber-reinforced concrete research. The keywords analysis shows that fibers-reinforced concrete having coconut fibers has been primarily explored for mechanical properties such as compressive and tensile strengths. The visualization map of keywords for co-occurrences, linkages, and the density related to the frequency of occurrence is presented in Figure 8. The keywords frequency is represented by circle size for a specific keyword, whereas the position of the circle depicts their co-occurrence in articles (Figure 8a). It can be observed from the map that leading keywords have larger circles showing their importance in research on coconut-fiber-reinforced concrete. The group creation is also performed for keywords to display the co-occurrence for numerous research publications. The color-coded groups are formed based on the co-occurrence of various keywords in published papers. The four colors that show the existence of groups are red, green, blue, and yellow (Figure 8a). However, Figure 8b depicts the density concentrations for keywords by using different colors. The assigned colors are based on their respective density concentration. Blue and red colors, respectively, show the lowest and highest density concentrations. Fibers, reinforced concrete, and compressive and tensile strengths have considerable density concentrations, as demonstrated by red symbols. Such outcomes would assist determined scientists in choosing keywords that identify the specific published data in a particular area.

### 3.4. Authors

**Figure 9:**
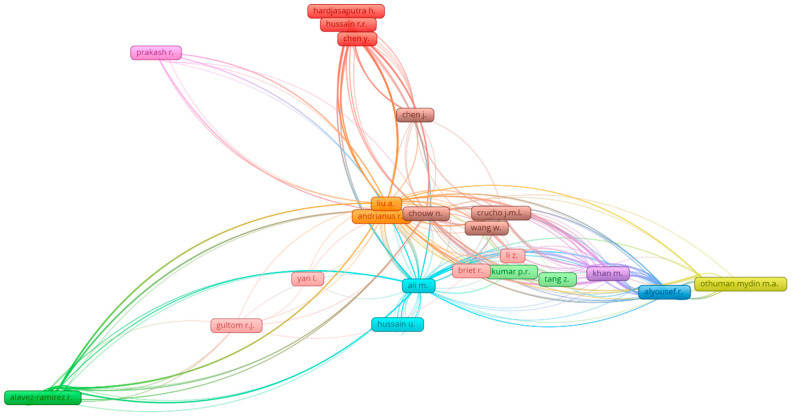
Scientific visualization of authors that published articles in the related research area.

The number of citations depicts the influence of that particular researcher in a specific research area [71]. Hence, “kind of analysis” is opted as “co-authorship”, and “unit of analysis” is selected as “authors” for the evaluation of authors. The criteria for the least articles are limited to five, and there are twenty authors with set criteria (Table 3). The researcher’s effectiveness is difficult to evaluate by taking all parameters, such as the number of publications and citations. Therefore, the assessment of a researcher is made in view of every parameter independently. The researcher having the most publications (i.e., 18 published articles) in the considered research area is “Chouw N.” followed by 17 published articles by “Ali M.”, whereas, as far as total citations are concerned, Ali M. leads this specific field having 600 citations followed by Chouw N. with 569 citations and Khan M. with 216 citations in the research area of coconut fiber in concrete. Figure 9 shows the correlation between the most distinguished authors and researchers having at least two publications. The observed most prominent interconnected researchers’ network is ten. This analysis reveals that some authors are inter-linked in citations for the coconut-fiber-reinforced concrete research area.

### 3.5. Documents

**Figure 10:**
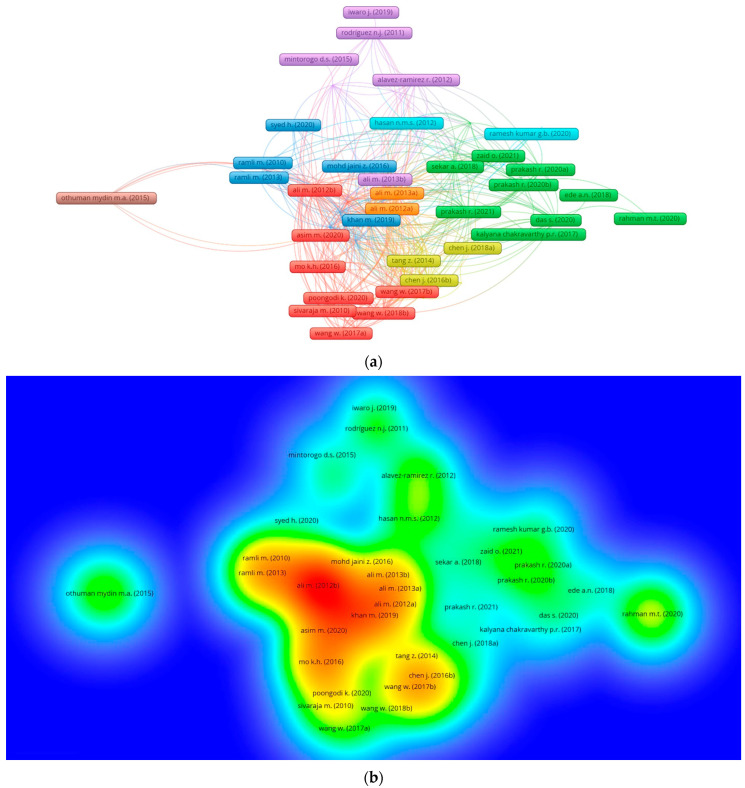
Scientific mapping of published articles in the related research area up to 2022: (**a**) Connected articles in terms of citations. (**b**) Density of connected articles.

A specific research area is usually influenced by the quantum of citations for an article. The articles with more citations are recognized as pioneers in that particular research area. To assess articles, “kind of analysis” is set as “bibliographic coupling”, whereas “unit of analysis” is adopted as “documents”. An article with at least 30 citations is set as a limit. In the coconut-fiber-reinforced concrete research field, the leading ten articles concerning citations are listed in Table 4. Ali et al. [72] have the most citations, 221, for the article titled “Mechanical and dynamic properties of coconut fiber reinforced concrete”. Ramli et al. [41] and Khan and Ali [73] have 96 and 81 citations, respectively, for their respective articles and lie in the first three positions. Moreover, Figure 10 depicts the mapping of linked articles, as per their density and citations in the under-studied area of research. Figure 10a shows the mapping of inter-related articles citation, and the density concentration enhancement by top articles is extracted from the density mapping, as revealed in Figure 10b.

### 3.6. Countries

**Figure 11:**
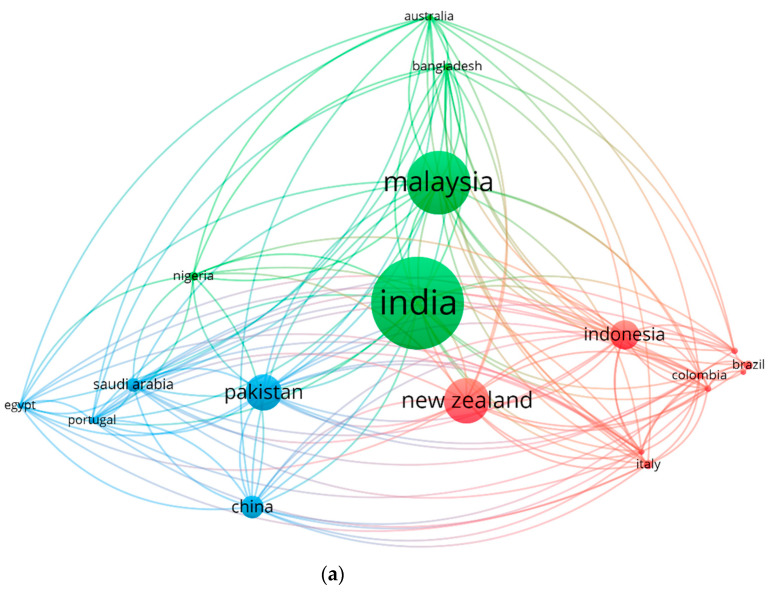

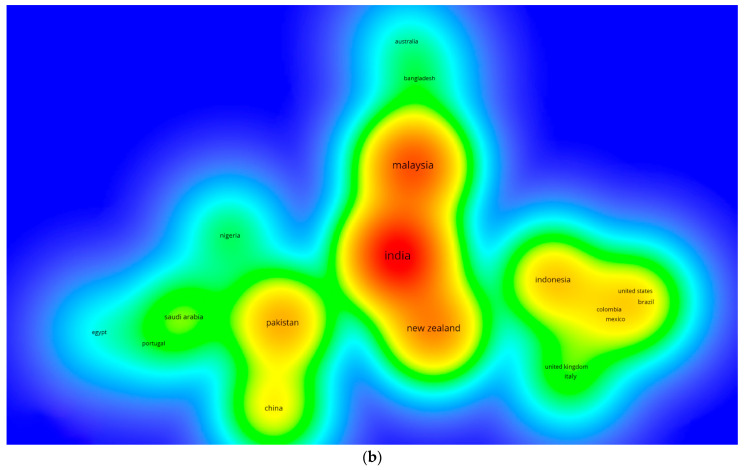
Scientific visualization of countries in the related research area up to 2022: (**a**) Network visualization. (**b**) Density visualization.

The involvement of numerous regions/countries is higher toward coconut fiber in concrete research, and there is still potential for increasing this contribution. The network map is established to aid scientists in accessing coconut-fiber-reinforced concrete research areas. Here, “kind of analysis” is set as “Bibliographic coupling”, and “unit of analysis” is designated as “countries”. The least number of articles per country is set at two, and Table 5 provides all the 38 countries with the desired criteria. The top 3 countries in this regard are India, Malaysia, and New Zealand, having 41, 28, and 20 articles, respectively. Moreover, the three leading countries as per research-area-based citations are New Zealand, Malaysia, and Pakistan, having 603, 473, and 367 citations, respectively. Figure 11 presents the mapping of science visualization along with citations inter-relation with density. The circle size depicts the impact of that specific country in that particular research area (Figure 11a). The density visualization map in Figure 11b shows that a higher density is for the most engaging countries. The statistical and graphical analysis of participating countries may assist the researchers of coconut-fiber-reinforced concrete areas to have scientific collaborations and alliances, and novel methods and ideas may also be exchanged among them.

## 4. Discussion and Future Perspectives

In this work, the statistical and mapping overview of diverse aspects of the available literature on coconut-fiber-reinforced concrete is presented. The manually conducted conventional review research has limited comprehensiveness and a less accurate inter-relation between different segments of the literature. Further, in this study, the assessment of journals having the most published articles, the most widely used keywords in published articles, the main contributing countries, and articles and authors having the most citations in the coconut-fiber-reinforced concrete research field is carried out. The keywords analysis reveals that coconut-fiber-reinforced concrete has primarily been discovered for its mechanical properties [42,45,72,73,75,76]. Furthermore, using conventional materials consumes the natural resources and energy at a larger scale, and the required processes emit bulk CO_2_ emissions [11]. Accordingly, concerns are rising about saving natural resources from excessive depletion. Thus, incorporating coconut fibers in concrete would decrease the cement and aggregates requirement, ultimately causing sustainable/green construction to have lower CO_2_ emissions [26,34,75,79].

It is well known that concrete is basically a majorly utilized material in the construction industry worldwide. However, in natural/conventional form, concrete lacks in strain capacity and resistance against tensile loading and cracking, and behaves more brittle [9,80,81,82]. In order to resolve these problems, the incorporation of fibers such as synthetic fibers [11], metallic/steel fibers [83], and natural fibers [45,84,85,86] in concrete is carried out for enhancing its toughness. Among all fibers, the addition of coconut fibers in concrete is beneficial to enhance the ductility under different types of loading (compression, splitting-tensile, and flexural), as required to obtain structural safety [73]. The addition of coconut fibers can decrease concrete’s brittleness in advance to improve various mechanical properties such as enhanced capability of energy absorption and significantly improved tensile strength. However, the coconut-fiber-reinforced concrete applications are still under development. In-depth studies are still needed before widening its applications toward structural members. Currently, the research on coconut-fiber-reinforced concrete primarily focuses on the insight into establishing the optimum mix design for improved properties. Still, there is a need to explore more horizons of using coconut fibers in concrete. The concrete workability with coconut fibers is lesser, which may be enhanced for an improved slump. Incorporating specific admixtures such as super plasticizers and air-entraining agents and pozzolanic materials such as fly ash should also be studied to enhance the concrete flow. Furthermore, the effect of the above-mentioned additives and silica fume should also be explored for improving the mechanical properties of coir-reinforced concrete under compressive, tensile, and flexural loadings. The manual mixing of coconut fibers in concrete is a tedious job that leads to a nonhomogeneous mix. In this scenario, the addition of certain chemicals may be studied to replace said hand-mixing with machine mixing. It is also important to explore the incorporation of coconut fibers on the cement matrix pore structure, crack abridgement, and chloride and water permeability characteristics of concrete. In addition, it is vital to establish an innovative strategy for developing coconut fibers’ water retention capability for high-performance concrete composites with the help of the internal curing process. Moreover, due to durability concerns due to the biodegradable nature of coconut fibers, the structural applicability of coconut-fiber-reinforced concrete at a larger scale is still limited. The comprehensive information on the life cycle assessment (LCA) of coconut-fiber-reinforced concrete is also insufficient and, hence, demands detailed exploration.

## 5. Conclusions

This research aims to conduct a scientometric analysis of the available literature in the coconut-fiber-reinforced concrete field for evaluating different measures. The 235 articles are assessed via the Scopus database, and the VOSviewer program is employed to determine the results. The conclusions made are as follows:

- The analysis of publication sources on coconut-fiber-reinforced concrete reveals that the leading three journals are “Construction and Building Materials”, “IOP Conference Series: Materials Science and Engineering”, and “Materials Today: Proceedings”, comprising 21, 9, and 8 articles, respectively. Moreover, the three leading journals regarding citations are “Construction and Building Materials”, “Materials”, and “Materials Today: proceedings”, having 876, 122, and 56 citations, respectively.
- The keywords analysis in the under-studied field of research shows that fibers, concretes, compressive strength, reinforced concrete, tensile strength, and coconut fibers are the six most widely appearing keywords. This analysis further reveals that fiber-reinforced concrete having coconut fibers is majorly explored for the construction industry.
- Based on the publications and citations, the leading authors are classified. Chouw N. and Ali M. are the two leading ones with 18 and 17 articles, respectively. Having 600 citations, Ali M. leads the field, followed by 569 citations from Chouw N. and 216 citations from Khan M. by 2022.
- The analysis of coconut-fiber-reinforced concrete-related articles shows that Ali et al. [72] have the most citations, 221, for the article titled “Mechanical and dynamic properties of coconut fiber reinforced concrete”. Ramli et al. [41] and Khan and Ali [73] have 96 and 81 citations, respectively, for their respective articles.
- The analysis for exploring the leading contributing countries is also conducted for the field of coconut-fiber-reinforced concrete research. India, Malaysia, and New Zealand have contributed 41, 28, and 20 articles, respectively. Moreover, the countries of New Zealand, Malaysia, and Pakistan have received citations of 603, 473, and 367 citations, respectively.
- Sustainable development can be attained by using coconut-fiber-reinforced concrete in the construction industry by declining CO_2_ emissions and saving natural sources.
- The applicability of coconut-fiber-reinforced concrete on a broader scale is still quite limited. Its structural applications are still under exploration due to limited knowledge of its long-term durability. Additional analysis on its long-term durability is also vital for widening the applications of coconut-fiber-reinforced concrete toward structural members.

## Figures and Tables

**Table 1:** Publication sources in the related research field up to 2022.

S/N	Publication Source	Number of Publications	Total Number of Citations
1	Construction and Building Materials	21	876
2	Materials	6	122
3	Materials Today: Proceedings	8	56
4	International Journal of Civil Engineering and Technology	6	35
5	Jurnal Teknologi	4	32
6	MATEC Web of Conferences	7	26
7	AIP Conference Proceedings	5	24
8	IOP Conference Series: Materials Science and Engineering	9	21
9	Key Engineering Materials	5	18
10	Scientific Reports	2	17
11	Case Studies in Construction Materials	2	10
12	International Journal of Mechanical Engineering and Technology	3	10
13	International Journal of Engineering and Technology (UAE)	2	7
14	Advances in Materials Science and Engineering	2	5

**Table 2:** The 30 leading frequently employed keywords in the research of coconut-fiber-reinforced concrete.

S/N	Keyword	Occurrences
1	Fibers	52
2	Compressive Strength	50
3	Reinforced Concrete	40
4	Concretes	35
5	Tensile Strength	30
6	Coconut Fibers	28
7	Concrete	27
8	Fiber-Reinforced Materials	26
9	Coconut Fiber	23
10	Cements	17
11	Mechanical Properties	15
12	Natural Fibers	15
13	Sustainable Development	14
14	Fly Ash	13
15	Aggregates	12
16	Coconut Fiber	12
17	Fiber Reinforced Concrete	12
18	Bending Strength	10
19	Concrete Aggregates	10
20	Construction Industry	10
21	Durability	10
22	Concrete Construction	9
23	Concrete Mixtures	9
24	Flexural Strength	9
25	Light Weight Concrete	9
26	Bridge Decks	8
27	Conventional Concrete	8
28	Fiber Reinforced Concrete	8
29	Agricultural Wastes	7
30	Coconut Shell	7

**Table 3:** Top 20 authors in the research of coconut-fiber-reinforced concrete up to 2022.

S/N	Author	Number of Publications	Total Number of Citations
1	Ali M.	17	600
2	Chouw N.	18	569
3	Khan M.	5	216
4	Ramli M.	3	142
5	Wang W.	6	106
6	Aslam F.	4	74
7	Othuman Mydin M.A.	8	74
8	Chen J.	5	62
9	Prakash R.	3	53
10	Raman S.N.	3	53
11	Subramanian C.	3	53
12	Thenmozhi R.	3	53
13	Ahmad J.	3	26
14	Khedher K.M.	3	26
15	Gunasekaran K.	3	15
16	Mydin M.A.O.	3	14
17	Lumingkewas R.H.	3	10
18	Singh J.	3	10
19	Lv Y.	3	7
20	Hadiwardoyo S.P.	3	6

**Table 4:** Top 10 highly cited published articles up to 2022 in the research of coconut-fiber-reinforced concrete.

S/N	Article	Title	Total Number of Citations Received
1	Ali, et al. [72]	Mechanical and dynamic properties of coconut fibre reinforced concrete	221
2	Ramli, et al. [41]	Strength and durability of coconut-fiber-reinforced concrete in aggressive environments	96
3	Khan and Ali [73]	Improvement in concrete behavior with fly ash, silica-fume and coconut fibres	81
4	Wang and Chouw [74]	The behaviour of coconut fibre reinforced concrete (CFRC) under impact loading	60
5	Ali and Chouw [75]	Experimental investigations on coconut-fibre rope tensile strength and pullout from coconut fibre reinforced concrete	51
6	Khan and Ali [45]	Effect of super plasticizer on the properties of medium strength concrete prepared with coconut fiber	50
7	Ahmad, et al. [76]	Effect of coconut fiber length and content on properties of high strength concrete	48
8	Ali, et al. [77]	Capacity of innovative interlocking blocks under monotonic loading	39
9	Ali, et al. [78]	Dynamic response of mortar-free interlocking structures	36
10	Majid [36]	Coconut fibre—A versatile material and its applications in engineering	36

**Table 5:** Leading countries based on published documents in the present research area until 2022.

S/N	Country	Number of Publications	Total Number of Citations
1	India	41	265
2	Malaysia	28	473
3	New Zealand	20	603
4	Pakistan	16	367
5	Indonesia	13	46
6	China	10	225
7	Saudi Arabia	6	109
8	Brazil	4	21
9	Italy	4	11
10	Nigeria	4	39

## Data Availability

Not applicable.
